# Job retention vocational rehabilitation for employed people with inflammatory arthritis: adaptations to the WORKWELL trial due to the impact of the COVID-19 pandemic

**DOI:** 10.1186/s13063-022-06941-2

**Published:** 2022-12-20

**Authors:** Angela Ching, Jennifer Parker, Alexandra Haig, Chris J. Sutton, Sarah Cotterill, Denise Forshaw, June Culley, Alison Hammond

**Affiliations:** 1 Centre for Human Movement and Rehabilitation, University of Salford, Salford, Greater Manchester UK; 2 Lancashire Clinical Trials Unit, University of Central Lancashire, Preston, UK; 3 Centre for Biostatistics, School of Health Sciences, The University of Manchester, Manchester, UK; 4 Patient Research Partner, Derbyshire, UK

**Keywords:** COVID-19, Pandemic, Randomised controlled trial, Trial management, Remote intervention delivery, Arthritis, Vocational rehabilitation, Occupational therapy, Work

## Abstract

There are high levels of work disability, absenteeism (sick leave) and presenteeism (reduced productivity) amongst people with inflammatory arthritis. WORKWELL is a multi-centre, randomised controlled trial of job retention vocational rehabilitation for employed people with inflammatory arthritis. The trial tested the effectiveness and cost-effectiveness of the WORKWELL programme compared to the receipt of written self-help information only. Both arms continued to receive usual care. In March 2020, due to the COVID-19 pandemic, the WORKWELL trial paused to recruitment and intervention delivery. To successfully re-start, protocol amendments were rapidly submitted and changes to existing trial procedures were made. The WORKWELL protocol was adapted in response to both the practical issues likely faced by many clinical research studies active across NHS sites during the pandemic and additional trial-specific challenges. A key eligibility criterion for the trial required participants to be in paid work for at least 15 h per week. However, UK national lockdowns led to a substantial proportion of the workforce suddenly being furloughed or unable to work, and many people with arthritis taking immunosuppressive medications were asked to shield themselves. Thus, the number of eligible participants was reduced. Those continuing to work were harder to identify, as hospital clinics moved to remote delivery, and also to then screen, consent and treat, as the hospital research staff and clinical therapists were re-deployed. New recruitment and consent strategies were applied, and where sites had reduced capacity, responsibilities were absorbed by the trial management team. Remote intervention delivery and electronic data capture were also implemented. By rapidly adapting the WORKWELL protocol and procedures, the trial successfully reopened to recruitment in July 2020, only 4 months after the trial pause. We were able to achieve recruitment figures above the pre-COVID target and maintain a high retention rate. In addition, we found many of the protocol changes beneficial, as these streamlined trial procedures, thus improving efficiency. It is likely that many strategies implemented in response to the pandemic may become standard practice in future research within trials of a similar design and methodology.

**Trial registration:** ClinicalTrials.gov NCT03942783. Retrospectively registered on 08 May 2019. ISRCTN Registry ISRCTN61762297. Retrospectively registered on 13 May 2019.

## Background

The COVID-19 pandemic has affected clinical trial activities, and there has been a huge shift in how clinical trials are managed [1]. Globally, most sites conducting non-COVID-19 research experienced trial pause and delay timelines [1]. Here, we describe the impact of the COVID-19 pandemic on the WORKWELL trial. This paper is split into three parts: (1) describes the WORKWELL trial and the original recruitment and intervention delivery procedures, (2) discusses the impact of COVID-19 on trial delivery and the adaptations made to recruitment and intervention delivery and (3) provides recommendations for future research.

### Part 1: The WORKWELL trial and the original recruitment and intervention delivery procedures

Work problems are common in people with inflammatory arthritis (IA). This affects about 1% of the United Kingdom (UK) population, causing joint pain and fatigue [2–4]. A third stop working due to their condition (work disability) within 5 years and 50% within 10 years of diagnosis [5]. Up to 67% report presenteeism (reduced work productivity), even amongst those with low disease activity [6]. Job retention vocational rehabilitation (JRVR) can potentially prevent or postpone work disability and reduce presenteeism through structured work assessment, work-related rehabilitation and modifying work demands to better match the person’s condition and abilities [7]. In the UK, many employed people with IA lack access to JRVR services. Work-related support in many rheumatology services within the UK National Health Service (NHS) is either non-existent or patchy [8].

The WORKWELL trial is a definitive, pragmatic, multicentre superiority randomised parallel-group trial recruiting employed people diagnosed with rheumatoid arthritis (RA), undifferentiated inflammatory arthritis (UIA), or psoriatic arthritis (PsA) who have work instability (i.e. a mismatch between their functional abilities and the demands of their job [9]). The trial is testing the effectiveness of a work intervention (i.e. the WORKWELL JRVR programme, written work self-help information pack and usual care) compared to a control group who receive a written work self-help information pack and usual care.

The primary outcome is the Work Limitations Questionnaire-25 (WLQ-25) at 12 months, which is a valid measure of presenteeism indicating the amount of time; in the last 2 weeks, a person was limited in physical work demands, time demands, mental-interpersonal demands and output demands [10]. Presenteeism is the most relevant primary outcome as it has the greatest impact on costs for people with RA [11]. Outcomes were collected at baseline and 6 and 12 months post-randomisation.

#### Original recruitment procedures (Fig. 1)

**Fig. 1:**
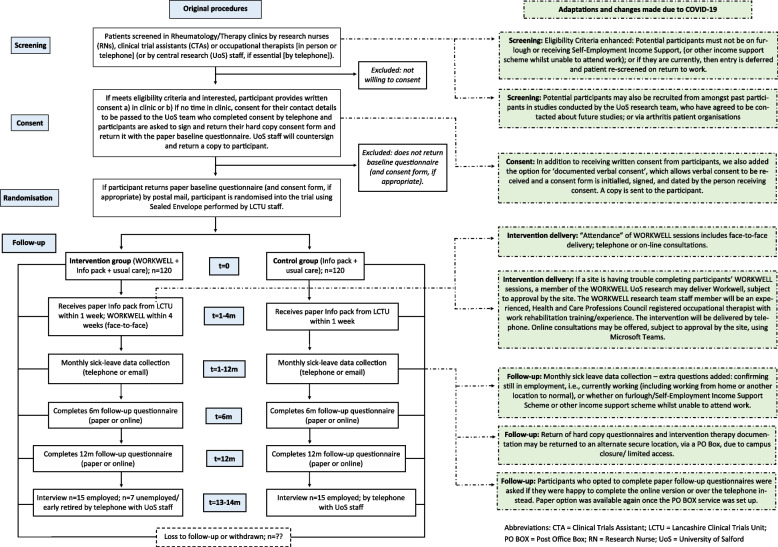
Flowchart of the original procedures and adaptations made

##### Screening and consenting

In the UK, people with RA, UIA and PsA are predominantly treated in rheumatology departments in secondary care. We identified, screened and recruited potential participants from hospital rheumatology or therapy outpatient clinics. Twenty-two hospitals across 18 NHS Trusts in England, Wales and Scotland in the UK were recruiting sites. The key inclusion criteria were aged ≥ 18 years; rheumatology consultant diagnosed RA, UIA or PsA; in paid work for at least 15 h per week; not on sick leave for more than 4 weeks; RA-Work instability Scale score of ≥ 10 (indicative of medium-to-high risk of work instability [9]); and able to attend for WORKWELL appointments. For the full list of the inclusion and exclusion criteria, please refer to the published WORKWELL protocol [8]. Research nurses, clinical trial assistants (CTAs) or occupational therapists at participating sites and the research team at the University of Salford (UoS) were involved in screening and consenting potential participants. (Occupational therapists at sites were those who were trained in delivering the intervention.) Participants provided written consent in the clinic with the research nurse, CTA or occupational therapist or consented to their contact details being passed to the UoS research team, who then completed screening and consent by telephone.

The target sample size was 180 participants with primary outcome data. Allowing for a 25% attrition rate, we intended to recruit and randomise 240 participants (intervention: 120 and control: 120) to achieve 90% power to detect a clinically important effect. Participants were randomly assigned 1:1 to WORKWELL intervention or control using ‘Sealed Envelope,’ a secure, online, central randomisation service [12]. For further details on the sample size calculation and randomisation process, see the published WORKWELL protocol [8].

Trial management was conducted by a joint team. The University of Salford (UoS) team (trial manager, trial administrator and chief investigator) was responsible for obtaining Health Research Authority (HRA), ethics and trial amendment approvals and confirmation of capability and capacity (C&CC) from research sites; conducting site liaison and site training and monitoring visits; providing remote recruitment for sites with no research nurse support; co-ordinating recruitment procedures and baseline postal questionnaire collection; arranging data collection from therapists; and site close-out. The Lancashire Clinical Trials Unit (LCTU: University of Central Lancashire) advised on the ethics application, amendments, documentation and all trial management procedures. The LCTU was responsible for randomisation, monitoring WORKWELL treatment progress, developing and managing online questionnaires and databases, monthly sick leave and all follow-up data collection; missing and minimal data set collection; and all data entry, verification and preparing data for the statistics team. The LCTU also managed the Study Within a Trial (SWAT) evaluating the effect of pre-notification letters on response rates [13]. The trial methodologist (CS) and lead trial statistician (SC) at the University of Manchester worked closely with the team advising on procedures.

#### Original intervention delivery procedures

Participants randomised to the intervention arm received usual care plus an individualised programme of JRVR to meet their priority work-related needs, as assessed using the UK Work Experience Survey-Rheumatic Conditions (WES-RC) [14]. According to the trial protocol, JRVR was delivered by NHS therapists who specialise in rheumatology and musculoskeletal conditions, with at least 2 years’ experience in rheumatology. The participating therapists were all occupational therapists. Unfortunately, those physiotherapists recruited to support the trial were unable to continue to do so because: their site withdrew prior to trial start (e.g. staff changes meaning the site no longer had capacity); therapists withdrew due to changed circumstances (e.g. job change, maternity leave); or therapists were unable to attend one of the three WORKWELL training courses available (during a 2-month period). Participating therapists attended one of the 2-day WORKWELL training courses, delivered by expert JRVR therapists (trainers) and the UoS research team. The training consisted of trial background; key study procedures; how to conduct the WES-RC; case studies used to identify key work problems and developing treatment plans with relevant work solutions; practical workshops (e.g. office seating and ergonomic equipment, manual handling, ergonomic options for tools); and self-study materials (e.g. analysing work activities). The therapists then completed a role-play telephone WES-RC with one of the JRVR trainers, based on a case study, and wrote an appropriate treatment plan, in order to demonstrate competency to deliver JRVR. Additionally, the trainers provided ongoing mentoring to the treating therapists during the treatment phase, answering queries about cases and advising on solutions. More details on therapists’ training are available in our published protocol [8].

WORKWELL therapists delivered the JRVR in up to four 1-h face-to-face appointments, held approximately monthly, and a 30-min telephone review 6 weeks later to discuss progress. Appointments were at mutually agreed times at participants’ usual outpatient rheumatology therapy department, with flexible appointments available earlier and later in the day to reduce time off from work to attend. Treatment was supported by the WORKWELL Work Solutions Manual, created for the trial, which aided therapists in developing practical solutions to work problems identified. Therapists were asked to record in their participants’ treatment notes the three priority work problems collaboratively identified with a participant and the solutions identified to address these: treatment plans, treatment delivered by the therapist related to each problem area and participants’ actions (e.g. requesting recommended job accommodations at work, making behavioural changes at work, such as pacing, performing tasks differently) and their responses to treatment. Treatment fidelity was then to be assessed by evaluating an audio recording of each therapist conducting the WES-RC with one participant and a review of the accompanying treatment notes for that participant. Further details on treatment fidelity are available in our process evaluation protocol [15].

#### Original follow-up data collection procedures (Fig. 1)

For the 6-month and 12-month follow-up questionnaires, participants could choose if they wanted to complete the paper or online version of the questionnaire. Monthly sick leave data was collected by the LCTU staff by telephone or email (participant preference). The data collected included (1) number of days of sickness absence attributable to ill health and (2) if participants were still in employment, and if not, the date they stopped work (or if not working, the date they re-started work). Telephone, text and e-mail reminders were used to maximise the return of questionnaires and sick leave data. Trial administrators at the LCTU telephoned/texted/e-mailed (as applicable) participants to obtain missing data and try to ensure a minimal data set (i.e. the primary outcome measure) was obtained from any non-responders.

As part of the process evaluation, approximately 15 participants from the intervention group and approximately 15 from the control group were interviewed by a member of the UoS research team by telephone after they completed their 12-month follow-up questionnaire.

### Part 2: Impact of COVID-19 on the WORKWELL trial

In this section, we describe the impact COVID-19 had on the trial and the actions we took from March 2020 at the start of the pandemic through to the trial re-start in July 2020 and onwards.

#### March 2020—COVID-19 pandemic and lockdown

The COVID-19 pandemic had a severe effect on non-COVID-related clinical research across NHS sites in the UK [16]. In mid-March 2020, many studies were paused to recruitment and treatment for patient and staff safety, and many NHS out-patient appointments were cancelled or not scheduled. In the NHS, research nurses and clinicians from different healthcare professions were redeployed to front-line work or nationally prioritised COVID-19 studies [16]. Many of the WORKWELL occupational therapists were redeployed. Additionally, from mid-March 2020, the trial management team at both universities had to rapidly switch to working from home, without access to office facilities or University mailrooms to send or receive postal questionnaires.

##### Revising the protocol

In early March 2020, the trial management team prepared a risk assessment for the Trial Management Group (TMG) and Trial Steering Committee (TSC) identifying potential problems due to the COVID-19 pandemic and potential solutions. In mid-March 2020, the TMG paused the trial to recruitment and treatment for patient and staff safety reasons and sites were notified. The collection of follow-up questionnaires continued. The trial management team and TMG identified mitigating actions for pause and re-start. Some potential issues anticipated at trial re-start were that many sites would have difficulty screening and obtaining written consent from participants to target, e.g. due to continuing staff redeployment. Another major concern was regarding participants already in treatment. Some sites might no longer be able to treat ongoing or new participants or not resume treatment until later. However, treatment had to resume as soon as possible, as it had to finish before the participants’ scheduled 12-month follow-up. Remote recruitment and treatment procedures were discussed and agreed upon as feasible with the TMG and TSC. We will discuss these changes later in this article.

#### April 2020—formal trial pause

In early April 2020, we formally notified trial pause to the Health Research Authority (HRA) and sites through a non-substantial amendment (category C), along with amendments to participant communications and questionnaire changes. We applied for and successfully received a 7-month no-cost extension from our funder, Versus Arthritis, allowing extra time for recruitment to meet our target (*n* = 240). A further non-substantial amendment (category C) was submitted in June 2020, with changes for trial re-start. These changes (discussed below) were approved by the sponsor (University of Salford) under HRA guidelines [17].

##### Trial progress at pause

At the time of the trial pause, 187 participants had been consented, of whom 162 had completed baseline questionnaires and been randomised into the trial (68% of the target). Of those in the intervention arm, 22 had completed treatment and 59 were in treatment (12 of whom were waiting to start treatment at the time of the trial pause). There were also participants: waiting to be randomised, who had either just returned or were in the process of returning, baseline questionnaires, or patients waiting to be screened or consented.

##### Actions with prospective and active trial participants

Early communication with prospective and active participants was essential to explain about the trial pause, and then actions at re-start, depending on their stage in the trial. We worked with our Patient and Public Involvement group to develop letters/e-mails informing:

1. Potential participants awaiting screening by the trial management team: to would be contacted again at trial re-start to be screened.
2. Participants provided with a baseline questionnaire but not yet returned: not to complete/return it (as it could not be retrieved from the University mailroom due to campus closure). We would contact them at re-start to request completing a new questionnaire.
3. Participants who had already returned their baseline questionnaire but not yet randomised: would need to complete a new baseline questionnaire at re-start, as their original questionnaire responses may no longer be valid due to changes in their health or work abilities.
4. Participants in the intervention group referred to occupational therapy for WORKWELL JRVR but who had not yet received their first appointment (delayed intervention participants: *n* = 12): would first need to complete a new baseline questionnaire, as their original responses may no longer be valid, but would then be re-referred to occupational therapy and receive treatment. (The TMG agreed the follow-up timelines for these participants would be calculated from their new baseline questionnaire return date.)
5. Matched control delayed participants (*n* = 12): as 12 intervention participants were delayed (see above), we also delayed 12 recently randomised control participants matched with the intervention group participants as closely as feasible by job skill level (a stratification factor in the randomisation). These participants were also informed they would be asked to complete a new questionnaire at re-start (with follow-up timelines calculated from the date of return).
6. Intervention participants who had already started the WORKWELL programme with an occupational therapist: treatment was suspended but would resume when their site confirmed re-start. Their follow-up continued as scheduled.
7. Control group participants, and those intervention group participants who had completed treatment prior to trial pause, were informed of the trial pause and that follow-up would continue as scheduled.

We contacted participants in groups 1, 2 and 7 by post/email, and we telephoned participants in groups 3, 4, 5 and 6 to explain the situation. We provided up-to-date information about COVID-19, arthritis and work from reliable sources 16 [18, 19] through our trial website (www.workwelluk.org). We informed participants about this and, on trial progress, through the regular trial newsletters.

#### July 2020 and onwards—trial re-start

The WORKWELL trial re-started at sites from July 2020 through to January 2021, as sites deemed they had the capacity and capability to re-start. However, rheumatology outpatient services and research support had radically changed. Many research nurses were still redeployed to frontline services or to work on COVID-19-related studies, and some treating therapists were still redeployed to other NHS services. For those clinical staff who returned to pre-COVID posts, the emergence of remote rheumatology and occupational therapy appointments caused challenges in identifying potential participants. For therapists, backlog, longer waiting lists and remote delivery increased service delivery pressures, which impacted their ability to ringfence time for recruitment and intervention delivery. Changing service demands, as COVID-19 hospital admissions rose and fell, meant NHS research staff and therapists could still be redeployed after trial re-start. The staff could also be on sick leave themselves due to COVID-19. We had to review the recruitment procedures and re-design the WORKWELL programme as appointments needed to be conducted remotely by occupational therapists, either by telephone or video, until deemed safe to resume face-to-face appointments.

##### COVID-19: impact on patients’ employment status and their eligibility in the WORKWELL trial

As mentioned in part 1, a key trial inclusion criterion was that participants be currently ‘at work’ (including working from home) for at least 15 h per week. However, national COVID-19 restrictions on social movement, including lockdowns, meant many potential participants were no longer able to be ‘at work’. Employed patients placed on the UK Government’s Coronavirus Job Retention Scheme (furlough), or claiming Self-Employment Income Support, became ineligible for the trial. Other potential participants were shielding, as ‘clinically extremely vulnerable’, e.g. if taking immunosuppressive or biologic medications, and strongly recommended by the UK Government and their Rheumatologists not to go to their workplace, even if essential workers. They remained eligible for the trial if working from home. Those unable to work from home, were also furloughed and so became ineligible. For those already participating in the trial, COVID-19 restrictions could lead to temporary redeployment, working from home or being furloughed at trial re-start.  For those in the intervention group,  treatment plans had to be adjusted accordingly. This situation continued following re-start. Lockdowns and restrictions on movement continued on and off following the trial re-start (July 2020) until February 2022. Workers who could work from home did so, but others were furloughed again or lost jobs due to business failures. The individual circumstances of participants had to be considered when adjusting the WORKWELL protocol to keep the study running.

#### Changes and adaptations to the WORKWELL trial due to COVID-19

In this section, we discuss the changes made to the WORKWELL trial protocol enabling successful re-start from July 2020. Firstly, we describe the practical changes to trial management: trial documentation, staff training on remote screening and recruitment procedures, remote data collection, follow-up procedures and remote site closure procedures. Secondly, the changes to intervention delivery, additional therapist training, remote consultations and intervention delivery, and changes to treatment fidelity data collection.

#### Changes to trial management

##### Changes to trial documentation

Some trial documentation had been provided only as hard copy or non-editable versions. Documents were adapted to be completed in Microsoft Word, with options of checkboxes, free text boxes and inclusion of electronic signatures. This made it easier for the staff working remotely to complete documentation without requiring scanners, printers, or photocopiers.

##### Recruitment training for site staff

All training was conducted remotely via Microsoft Teams to reduce infection risks. Prior to re-start at each site, the trial management team held detailed online meetings with each site, with the principal investigator, research nurse/CTA (if available) and occupational therapist(s). During these meetings, we identified for each site what activities staff now considered feasible to continue with, ensured sites knew what was happening with each of their participants;  and explained the protocol changes to recruitment and treatment procedures and the new or amended documentation. Some sites could now only identify but no longer screen and consent participants or might be able to later dependent on how COVID-19 continued to impact services. Some sites could no longer treat ongoing or new participants or not resume until later. Re-start was negotiated and individualised according to what procedures were feasible at each site, with recruitment and treatment support from the trial management team tailored to meet each site’s needs. Principal investigators then applied to their Research and Development Department for trial re-start. Sites re-opened once confirmation for capacity and capability was obtained (between July 2020 and January 2021).

We also timetabled fortnightly drop-in online meetings (varying throughout the day) during the re-start period and continued individual ‘as and when’ e-mail access to the UoS trial manager, so that site recruiting staff could raise any queries. The UoS trial manager also recorded a presentation about the revised recruitment procedures, highlighting key changes due to COVID-19. This was available as a refresher and for site staff unable to attend re-start meetings, and accessible via a password-protected page on the trial’s website.

##### Changes to screening and recruitment: provision of trial management team support (Fig. 1)

A change to the trial inclusion criterion related to employment status was required. Patients were now ineligible if furloughed or on Self-Employment Income Support at the time of screening, as they were not ‘at work’, even though still employed. This was because our primary outcome measure (the WLQ-25 [8]) asked about abilities to work within the last 2 weeks, and these patients would be unable to answer this. However, if otherwise eligible, the recruiting staff (at sites or the trial management team, as appropriate) kept in contact with these patients to identify if they returned to work within the recruitment period: if they did, they were re-screened. We also clarified the screening procedures to the recruiting staff at sites, to explain that participants continuing to work from home, or from a different location or in a different role to normal, were still eligible.

In addition to the normal face-to-face consent procedure, we introduced documented verbal consent (DVC), allowing remote consenting by the trial management team or site staff. DVC form statements were read to the participant, each initialled and the form signed by the staff member receiving consent (Fig. 1).

At trial re-start (between July 2020 and January 2021 across sites), the combination of a lack of research nurses/CTAs to recruit, WORKWELL occupational therapists managing long waiting lists and most rheumatology and therapy out-patient appointments being delivered remotely meant it was more difficult for the site staff to identify and recruit participants. During the trial pause, we had recommended sites keep a list of any patients they considered could be screened at re-start. Occupational therapists reported that, between March and August 2020, this was difficult or impossible, as reasons for most referrals were different. Many patients were experiencing acute flares of their inflammatory arthritis or difficulty coping psychologically with the pandemic. As a result, they were not eligible as on sick leave, or they were too distressed to be approached about trial participation. Therapy priorities were to support urgent physical and mental health needs and keep patients out of the acute setting. Accordingly, we made changes to our recruitment strategies.

We already had the option in our trial protocol for the trial management team to support sites with remote telephone screening, recruitment and/or consent (with consent forms then completed by post), as required (Fig. 1). This was because the trial management team could contact patients during evenings and weekends, whereas most site staff could only do so during their core working hours (i.e. Monday–Friday, 8 am–5 pm, when many employed patients are at work), and some sites did not have research nurses/CTAs available to support recruitment. At re-start, we extended this support to all sites, as needed. The site staff still had to identify potential participants. Our protocol already allowed identification to occur through searching clinic databases, medical and therapy records, and some sites added this approach. The site staff still needed to provide introductory written trial information, and obtain patients’ documented verbal or written permission to allow them to forward their patients’ contact details to the trial management team. The trial team then contacted patients by telephone, explained the study, screened and, if appropriate, progressed to receive documented verbal consent, with a copy mailed to the participant and site for the patient’s records. There were benefits to these changes. Remote recruitment was easier following re-start as many potential participants were working from home, and telephone screening could be arranged at a convenient time for them, with DVC forms streamlining consent.

After re-start, it became clear that the recruitment target could not be met by solely relying on recruiting from NHS sites. There was not enough time or capacity to open new sites, which would probably have the same recruitment issues, and to train their therapists. Additional recruitment strategies were required. We therefore obtained HRA and University of Salford Ethics Panel approval to include volunteers in the trial. Firstly, the UoS research team held a volunteer database of employed people with RA, who had previously participated in UoS work-related research, and consented to being contacted about future studies (Fig. 1). As this previous work research had similar eligibility criteria to the WORKWELL trial, these volunteers were likely to be eligible for the trial, if still working. A number were originally recruited from sites which were now WORKWELL trial sites. We identified such volunteers, asked site staff to confirm they could be contacted (e.g. had not developed any health conditions which might preclude contacting them) and checked therapists were able to treat them. Secondly, we identified volunteers from non-WORKWELL sites. Both groups of volunteers were mailed trial information and asked to complete and return a contact details form to the UoS trial management team, if interested in taking part. Thirdly, we contacted the National Rheumatoid Arthritis Society (NRAS) and Versus Arthritis for assistance in identifying volunteers (Fig. 1). The trial management team devised short trial adverts, including key entry criteria, to circulate on NRAS’ and Versus Arthritis’ social media (Twitter/ Facebook) and websites. Both charities were able to provide a list of volunteers responding to the adverts. NRAS has a team facilitating recruiting to studies for a negotiated fee. Volunteers were screened by the trial management team. From those identified by NRAS and Versus Arthritis, we added additional questions to help confirm they had inflammatory arthritis: which medication(s) they were taking (as being on disease-modifying anti-rheumatic or biologic drugs is indicative of a diagnosis of inflammatory arthritis) and which Rheumatology NHS outpatient department they attended, and their consultant rheumatologist’s name (as people with inflammatory arthritis should be under the care of a Rheumatologist). These volunteers would be treated by the UoS WORKWELL research staff (see the ‘Intervention delivery’ section and Table 1).

**Table 1:** Intervention delivery: original plans and adaptations made due to COVID-19

Intervention delivery	Original protocol	Adaptations made due to COVID-19
Training for occupational therapists	• Two-day in-person WORKWELL training course • Role-play telephone WES-RC with one of the JRVR trainers • Receive mentor support by telephone and email from one of the JRVR trainers • WORKWELL Solutions Manual, available in hard copy	• Therapists were contacted to identify if they considered it feasible to and if they were confident in delivering WORKWELL remotely. Consensus was yes, as therapists had already changed service provision to be remote • Additional training in remote delivery was provided: the British Society of Rheumatology Guidelines for Remote Consultations, best-practice advice from the British Psychological Society [20], and other remote treatment resources; WORKWELL Solutions Manual, made available online plus additional video resource weblinks (e.g. setting up office chairs and home offices ergonomically [21], online exercise programmes to encourage people to keep moving, especially when working from home and during COVID restrictions [22, 23]) • Online training held with all therapists to explain protocol changes related to intervention delivery • Fortnightly timetabled online drop-in treatment discussion sessions added to increase the flexibility of access to mentors/other therapists to discuss treatment issues; e-mail discussion group for therapists to seek advice from each other • Therapists’ trial documentation (e.g. the WES-RC) made available as Word documents for electronic completion; further online resources for participants about returning to the workplace at the end of shielding or furlough and Rights at Work made available on the trial website; therapists provided with access to the online ‘SARAH’ hand exercise training programme for patients [24]
Work assessment	• WES-RC conducted face-to-face in rheumatology/therapy clinics • Optional work site visits	• WES-RC interview could be delivered remotely (telephone or video consultation) • Work site visits could be replaced by requesting participants email digital photographs of them in different working positions in relation to relevant equipment
Intervention delivery	• Up to four × 1-h face-to-face meetings and a 30-min telephone review after 6 weeks • One workplace visit, if appropriate	• Some sites could resume face-to-face delivery, using personal protective equipment, social distancing, and infection control • If face-to-face was not appropriate, then therapists could deliver the intervention by telephone or video consultation • Two WORKWELL research staff (employed at UoS) would deliver WORKWELL JRVR at any sites experiencing difficulties continuing to deliver WORKWELL JRVR • These two therapists also provided remote treatment to participants recruited from the volunteer database, NRAS and Versus Arthritis

*h* Hours, *JRVR* Job retention vocational rehabilitation, *min* Minutes, *NRAS* National Rheumatoid Arthritis Society, *SARAH* Strengthening and Stretching for Rheumatoid Arthritis of the Hand, *UoS* University of Salford, *WES-RC* Work Experience Survey-Rheumatic Conditions

##### Data collection and management

*Setting up a remote PO BOX delivery service (Fig.* 1*)*

Due to lockdowns, social movement restrictions or the need to reduce footfall on campuses, there were limited periods until February 2022, in which teams could access University research offices and mailrooms. By trial re-start (July 2020), a post office box (PO Box) delivery service with Royal Mail was set up so that post could be collected by the UoS trial manager. This allowed for the resumption of baseline questionnaire data collection (as these were only available in paper format) and offering paper 6- and 12-month questionnaire data collection options to those in follow-up. The PO Box also allowed site staff to post trial documentation to the research team, for those sites unable to e-mail electronic or scanned documents.

*Follow-up (Fig.* 1*)*

Throughout the pandemic, the LCTU continued to collect follow-up data without significant disruption or delay. University office closures meant we were initially unable to print, post or receive paper questionnaires for a short period. Questionnaire completion electronically was also preferred due to infection control requirements of handling paper questionnaires. At the point of consent into the trial, participants were always able to choose if they would prefer to receive online or paper follow-up questionnaires. As a result, all participants who previously chose to receive paper questionnaires were contacted by the LCTU research team and asked if they were able and willing to swap to an online questionnaire instead. For those that were unable to access the online questionnaire, or still chose not to, we offered telephone data collection until the PO Box facility became available to resume the use of postal questionnaires. Telephone data collection required significant time contributions from the participants and LCTU team. To reduce the burden on such participants, and to ensure key primary outcome data were collected, telephone follow-ups were arranged at times most convenient to participants. Also, questionnaire items were re-ordered to ensure core outcome data were collected first. The minimum core outcome dataset was collected from those who could not commit sufficient time to providing data for all outcomes.

To complete our primary outcome measure (the WLQ-25), participants needed to have worked (including working from home) at least 1 day in the last 2 weeks. Additional instructions were included in the follow-up questionnaires to advise any participants not working in the last two weeks (e.g. due to being on furlough or Self-Employment Income Support, self-isolating due to COVID-19, strict social distancing or government requirement) when they should complete their questionnaire. Those anticipating resuming work within the next 4 weeks were told to delay completing the questionnaire until they re-started work and to inform the trial management team about this delay. For those anticipating not resuming work within 4 weeks, they were asked to complete and return the questionnaire, but omit completing the WLQ-25. This followed guidance we already had in place for participants receiving questionnaires when on sick or annual leave. The follow-up questionnaires already included an item asking if participants had stopped work in the last 6 months and the reason for this. An additional response option was included: ‘caused by the coronavirus pandemic (for example, employer made you redundant; your employer’s business or your business closed)’. The LCTU liaised with participants to determine their working status and advised them when to complete their questionnaires, ensuring that all those eligible were completing the primary outcome measure where possible.

Participant employment status and sick leave details were collected every month (for 12 months) by email or telephone call. The sick leave data collection process was not impacted by the pandemic and continued as normal. An additional question, asking if the participant’s employment status or working circumstances had changed due to the pandemic (e.g. furlough or working from home), was added to the monthly sick leave request (Fig. 1). As this data was collected more frequently and in advance of follow-up questionnaires, it was used to inform the number of participants likely to be eligible to complete the primary outcome. Therefore, we could estimate in advance retention rates for the 6- and 12-month follow-up. This was a useful tool in supporting discussions at the monthly Trial Operational Group meetings, regarding ongoing recruitment and retention, recruitment plans and whether more participants needed to be recruited to ensure the minimum required number of completed primary outcome measures was met.

##### Electronic files

Pre-pandemic, electronic and paper trial master and site files were utilised by the trial management team, and paper site files at NHS sites. As a result of the pandemic, we included in an ethics amendment the option for sites to switch to electronic files. Trial pause increased the number of amendments, document additions and changes required and therefore site file management by the site staff. Posting hard copies of amendment documentation could have caused challenges to sites, due to either lack of support from research nurses to maintain files or working in different locations without access to site files. Additionally, emailing documents for the site staff to print for filing was also a challenge, as some no longer had ready access to printing facilities.

##### Site closure

Due to the COVID-19 pandemic, conducting face-to-face monitoring was more challenging due to restrictions on travel, lockdowns, research nurse and therapist redeployment to different departments and/or hospitals, and the continuing priority for research nurses to work on COVID-19-related studies. Thus, we also submitted an amendment to allow the option of remote monitoring and close-out of the trial if the site’s PI, R&D and the trial management team deemed this more appropriate. The option of an electronic site file facilitated this. Electronic site files have been located on a site-specific Microsoft Teams page (or another more appropriate platform advised by site’s R&D), and access is provided only to the PI, trial-specific research nurses/CTAs and a relevant member of the site’s R&D team who were supporting monitoring, close-out and archiving.

#### Changes to intervention delivery

##### Training in remote WORKWELL delivery for occupational therapists

All training was conducted remotely via Microsoft Teams to reduce infection risks. Prior to trial re-start, all therapists had switched providing their normal rheumatology therapy service to remote consultations and were already building skills and identifying creative solutions in delivering services remotely. Most sites had already provided additional telephones and laptops, with headsets and cameras, to enable therapy departments to do so. For most, the only face-to-face service provision was splinting, when essential. However, much of this was also provided remotely by providing manufactured splints, based on measurements taken by patients, and verbally or visually remotely checking splint fit.

To support WORKWELL provision, prior to re-start, the UoS trial management team adapted the trial protocol and provided additional training and resources to WORKWELL therapists (Table 1).

##### Remote consultations

The initial work interview, the WES-RC, which was used to identify participants’ work barriers, was designed to be conducted either in person or by telephone [25]. As part of their JRVR training for the trial, all occupational therapists successfully role-played conducting a WES-RC telephone interview with one of the JRVR trainers acting as a patient. This included collaboratively identifying work barriers and agreeing treatment priorities (see the WORKWELL process evaluation protocol for more detail [15]). With feedback from therapists, we agreed the initial interview could be delivered remotely (Fig. 1 and Table 1). Participants were asked at the first appointment to complete a work activity diary before their next appointment, to provide further detail about job tasks and levels of pain and/or fatigue experienced during these. This could be e-mailed to participants and returned by e-mail to the therapist before the next meeting to enable discussion about changing work patterns and tasks. Thereafter treatment was individualised and could include, for example, fatigue and stress management, psychological support, explaining rights under the Equality Act and discussing disclosure at work. These strategies, relying on discussion, could be provided remotely. Occupational therapists normally provide some patient interventions and liaise with other agencies by telephone and are already familiar with this approach. However, practical strategies, such as discussing ergonomic equipment options, ergonomic position training, hand exercise training and hand splint provision, could be more challenging as these rely on visual information and physical as well as verbal feedback. We agreed that such interventions could be supported by e-mailing additional information and weblinks to the online video material to participants and asking them to read and view these in advance of and after treatment sessions, as applicable. New information could be shared online during a video consultation or e-mailed to a participant during a telephone appointment for them to open and view. All participants had provided e-mail addresses facilitating such contact. Optional work site visits could be replaced by requesting participants to e-mail digital photographs of them in different working positions in relation to relevant equipment (e.g. working in their home office set-up), subject to any employer approval and avoiding inclusion of any others’ identifying features (Table 1). The final treatment review meeting was normally conducted by telephone.

##### Intervention delivery

Sites varied as to what stage they were able to resume face-to-face delivery. Some could offer this at re-start, using personal protective equipment, social distancing and infection control (Table 1). However, NHS Trusts needed to reduce footfall in hospitals to reduce COVID-19 transmission risks, and many participants were on immunosuppressive medication. For patient and staff safety, we recommended sites provided remote delivery (Table 1). If in-person treatment was deemed essential by a therapist and/or participant for all or part of treatment, we had sufficient funds for taxi fares to avoid participants travelling on public transport.

Following re-start, we asked therapists to provide informal feedback via e-mail and in mentor sessions, to ‘sense check’ their experiences of delivering WORKWELL remotely, to help us understand if it was feasible and if any problems were arising (Table 2). Remote delivery was considered feasible and, on balance, often preferable, given that participants were all employed. As part of the process evaluation, after therapists completed delivering WORKWELL, the therapists and participants were formally interviewed about their views of providing/receiving WORKWELL face-to-face and/or remotely. These results will be reported separately.

**Table 2:** Feedback from therapists on their experiences of delivering WORKWELL remotely

Advantages of remote appointments	Limitations of remote appointments
• Reduction in ‘unable to attend’ and ‘did not attend’ rates • Reduced impact on participants’ working day: they could re-locate to a private area at work for a consultation then return straight back to work or easily fit appointments in whilst working from home • Increased therapy appointment slots • Valued by participants anxious about attending hospital during COVID-19; opportunity for therapists to build trust and confidence to engage anxious patients in therapy • Encouraged participants to take more responsibility for problem-solving/identifying work solutions, as participants needed to be more explicit during the interview and in explaining work activity diaries, which further raised their awareness of their work problems • Ongoing provision of trial treatment during the pandemic • Work issues addressed in a timely manner • Psychological support to participants in a timely manner	• Difficult if needed to conduct a hand assessment as participants’ interpretation of hand anatomy sometimes is confusing, e.g. ‘knuckles’ often classed as distal interphalangeal joints (rather than metacarpophalangeal joints) • Mentally draining for therapists and participants, as more demanding on concentration levels • Additional questioning and explanation required as unable to pick up non-verbal responses or provide demonstrations (telephone appointments). Therapists quickly learnt alternative questioning techniques • Potential problem if the participant is stoical, as it could be easy for them to underplay problems. Lack of non-verbal cues to help identify this • Patients did not always place the same value on the telephone call/telehealth and requested a call back as not a convenient time, despite receiving a formal appointment date and time • Less personal if dealing with sensitive issues, e.g. bereavement, family illness

We anticipated that therapists might have difficulties continuing to deliver WORKWELL JRVR and that capacity could also vary following re-start. We therefore included in a trial amendment that two WORKWELL research staff (employed at UoS) would deliver WORKWELL JRVR at any sites experiencing such difficulties (Fig. 1). Both were experienced rheumatology occupational therapists, familiar with the WES-RC and providing JRVR. These two therapists would also provide remote treatment to those participants recruited from the volunteer database (not from WORKWELL sites) and via NRAS and Versus Arthritis. We therefore obtained amended Letters of Access or honorary contracts for the two UoS therapists from participating NHS Trust Research & Development departments, along with site Principal Investigator agreements, allowing them to treat participants on the site’s behalf. Between them, the UoS therapists treated (partially or entirely) 16 NHS participants, as well as 14 volunteers (i.e. 24% of the intervention group).

##### Changes to treatment fidelity data collection

To allow for monitoring of treatment fidelity, WORKWELL therapists normally audio-recorded, using a Dictaphone placed between the therapist and participant, their first treatment session (i.e. conducting the WES-RC interview), with one of their intervention participants. The audio-recording was made available securely to the trial management team, along with a full set of that participant’s treatment documentation. Both recording and documents were then analysed for treatment fidelity. Following the COVID-19 restrictions, remote treatment delivery posed challenges to audio data collection. Many therapists lacked access to telephones, video platforms or computer equipment that were compatible with high-quality audio-recording and were sometimes conducting remote consultations in busy shared offices using headphones. Such treatment sessions could not be recorded using a Dictaphone, despite attempts to resolve this. We did not have ethical approval to obtain video recordings from NHS video platforms nor had participants consented to this. As a result, treatment fidelity assessments for therapists unable to audio-record could only be done through document review. More detail on the WORKWELL trial treatment fidelity processes can be found in the process evaluation protocol article [15].

### Part 3: Recommendations for future research

At the start of the pandemic, adaptations were made to the trial to ensure it could continue remotely. Discussion amongst the TMG on trial adaptations led to the following recommendations for designing and implementing future trials, outside the confines of national lockdown.

#### Intervention development

When developing and refining therapy interventions and programme theory, the feasibility, benefits and limitations of remote delivery should be discussed with participating therapists, patients with the health condition, public and other key stakeholders. The Framework for the Development and Evaluation of Complex Interventions provides guidance for how to do this [26]. At the start of the WORKWELL trial, remote delivery would not have been feasible across all sites, due to insufficient IT facilities, and lack of therapists’ experience providing remote treatment, which would likely have reduced the number of sites willing to participate. However, the COVID-19 pandemic has led to marked improvements in both.

#### Recruitment

E-recruitment and documented verbal consent forms, allowing electronic signatures, streamline and speed up the consent process, compared to using a paper and postal approach. These, or other online consenting methods, should be available at the trial start.

#### Intervention delivery

In this trial, insufficient access to IT facilities in many therapy departments at the trial start meant we did not initially consider providing remote delivery, e-case report forms or online training. However, the COVID-19 pandemic has rapidly increased the move to telemedicine and remote rehabilitation delivery, and improved IT facilities, meaning that this approach is now more acceptable and feasible for therapists and patients [27]. As a result, therapists remote delivery skills developed and using electronic documents became easier, especially for therapists working from home, and during remote appointments. Remote intervention may be preferable to many patients, e.g. those who are employed (reducing the length of time taken out of work), have mobility problems, live in remote areas, have limited access to transport, have problems parking at their hospital, have psychological difficulties affecting hospital attendance and limited finances to pay for travel.

Remote treatment delivery proved feasible for therapists and was reported as often preferred by participants. In our trial, this was especially beneficial as participants were employed. Offering this could also increase recruitment rates, as treatment would be perceived as more easily accessible by patients. Conducting online site visits and training became easier as the NHS rolled out Microsoft Teams in NHS Trusts, meaning this became accessible and familiar to site staff. Online or e-case report forms can make reporting easier for treating therapists who have adequate IT facilities at their NHS site. Feasibility will be influenced by the nature of the assessment and intervention, as well as therapists’ adequate access to laptops or tablet computers.

#### Follow-up and archiving

Although electronic reporting by trial participants had become increasingly commonplace, the COVID-19 pandemic refocused its practicality in many circumstances. Despite the increased usage and uptake of online questionnaires during the COVID-19 pandemic, it remains important to continue offering paper options in trials, particularly for those participants without access to computer facilities, good and affordable wireless Internet access or limited digital skills. This increases the inclusivity of a broad range of participants in trials and can help with understanding how the study findings translate into real-world applications [28].

Finally, electronic site files reduce the need for paper documentation. However, a better method would be an online platform allowing storage of site-specific files, to which trial management teams could upload new documentation, saving site staff time, and allowing regular checking that site files are up-to-date.

## Conclusions

The WORKWELL trial successfully overcame hurdles due to the COVID-19 pandemic (e.g. staff re-deployment and remote clinics), as well as trial-specific challenges. The eligible employed participant pool shrunk significantly, due to furlough and shielding schemes, impacting recruitment. These unexpected changes in employment status affected primary outcome measure completion rates, intervention delivery and treatment plans. By rapidly implementing protocol amendments, we were able to re-start the trial, continue to deliver the intervention, recruit to target and maintain a high retention rate.

The impact of trial pause/re-start and the extensive changes required to many aspects of trial management should not be underestimated [29]. The additional workload (e.g. to devise alternate procedures, implement trial amendments, support remote recruitment, treatment and alternate data collection procedures required due to pandemic restrictions) was challenging and led to significant increases in trial management staff time and therefore costs, which had to be absorbed in existing budgets, no-cost extensions and balanced with workload in other studies. As a medical charity-funded trial, the academic researchers worked additional unpaid hours. NHS staff had to learn new procedures and adopt these in challenging circumstances. Timely and good communication within and between the trial management team, TMG, TSC and all key stakeholders, along with the willingness to be flexible in the light of rapidly changing circumstances, are essential for effective trial management [30]. After we had made these changes, Guidance for Significant Major Study Events became available to help trial teams in the planning process [29]. This recommends contingency planning is included at the start of all trials [29].

Reflecting on the adaptions made in response to the pandemic, many new procedures proved effective at keeping the trial running throughout the COVID-19 pandemic. Furthermore, we demonstrated that remote intervention delivery and online data collection are feasible in this context, even outside of a pandemic. Many adaptations improved trial management efficiency, streamlined processes, enabled remote trial and data management and were cost-effective. If instituted from the start of trials, these would help reduce staff time and associated costs (e.g. postage, stationery, printing, travel). Importantly, such changes would also reduce the carbon footprint of trials in line with recommendations from the National Institute of Health Research [31]. Therefore, many changes made may become standard practice in future research within trials of a similar design and methodology.

## Data Availability

Not applicable.

## Abbreviations

CTAs: Clinical trials assistants
CTU: Clinical trials unit
JRVR: Job retention vocational rehabilitation
NHS: National Health Service
NRAS: National Rheumatoid Arthritis Society
PI: Principal investigator
PO Box: Post office box
R&D: Research & Development
RA: Rheumatoid arthritis
SARAH: Strengthening And Stretching for the Rheumatoid Arthritis of the Hand exercises
TMG: Trial Management Group
TSC: Trial Steering Committee
UK: United Kingdom
WES-RC: Work Experience Survey-Rheumatic Conditions
WLQ-25: Work Limitations Questionnaire-25
